# Patient reported outcome measures in spinal muscular atrophy and duchenne muscular dystrophy: review of instruments and their inclusion in clinical and regulatory processes

**DOI:** 10.1007/s10072-025-08600-1

**Published:** 2026-02-13

**Authors:** Francesco Malandrini, Clarissa Spataro, Michela Meregaglia, Valeria Sansone, Adele D’amico, Annalisa Scopinaro, Oriana Ciani

**Affiliations:** 1 SDA Bocconi School of Management, CeRGAS – Centre for Research on Health and Social Care Management, Via Roberto Sarfatti, 10, 20136 Milano, Italy; 2 Roche S.P.A., Monza, Italy; 3 Neurorehabilitation Unit, The NeMO Clinical Center, University of Milan, Milan, Italy; 4 Unit of Muscular and Neurodegenerative Disorders, IRCCS, Bambino Gesù Children’s Hospital, Rome, Italy; 5 UNIAMO Federazione Italiana Malattie Rare, Onlus, Italy; 6 Faculty of Pharmacy, Paris-Saclay University, GRADES, 17 Av. Des Sciences, 91400 Orsay, France

**Keywords:** Patient-reported outcome measures, Spinal muscular atrophy, Duchenne muscular dystrophy, Health-related quality of life, Drug development and regulatory processes

## Abstract

**Objectives:**

This study aims to analyze the use of patient-reported outcomes measures (PROMs), observer-reported outcome measures (ObsROMs), and caregiver-reported outcome measures (CROMs) in spinal muscular atrophy (SMA) and Duchenne muscular dystrophy (DMD). The objectives are twofold: (1) to identify and characterize available instruments to be used in research and clinical practice, and (2) to assess their inclusion in drug development and regulatory assessment processes.

**Methods:**

A systematic search was conducted using PubMed, Google Scholar, Scopus, and the ePROVIDE database to identify PROMs, ObsROMs, and CROMs for SMA and DMD. The identified instruments were analyzed for validation, psychometric properties, Minimal Clinically Important Difference (MCID), and recall period. Additionally, clinical trial protocols, relative study publications, European Public Assessment Report (EPAR), and Italian Medicines Agency (AIFA) reports for innovativeness recognition on medicines for SMA and DMD (i.e., nusinersen, onasemnogene abeparvovec, risdiplam, and ataluren) were reviewed to evaluate the inclusion of these measures in drug development and regulatory assessment.

**Results:**

The initial search identified 50 questionnaires, including 40 PROMs, 5 ObsROMs, and 5 CROMs. Of these, 15 (30.0%) instruments were included in pivotal clinical trial protocols, with none designated as primary endpoints. Only 6 (12.0%) instruments were mentioned in EPARs, and MCID determination was reported for 6 (12.0%) of the instruments. Generic instruments like the PedsQL were frequently used but criticized for limited specificity.

**Conclusions:**

Despite the availability of PROMs, ObsROMs, and CROMs for SMA and DMD, their use in clinical trials and regulatory documents is limited and inconsistent. Greater standardization and systematic inclusion of these measures are needed to support patient-centered drug development and evaluation.

**Supplementary Information:**

The online version contains supplementary material available at 10.1007/s10072-025-08600-1.

## Introduction

In the evolving healthcare landscape, the patient's perspective has gained unprecedented recognition as a crucial element in assessing the effectiveness and quality of medical interventions. In this context, patient-reported outcomes (PROs) are a type of Clinical Outcome Assessment (COA) that enables the standardized description and monitoring of several constructs, such as symptoms, functions, and health-related quality of life (HRQoL) [1]. The U.S. Food and Drug Administration (FDA) defines a PRO as "a measure of a patient's health status reported directly by the patient, without interpretation by a healthcare professional or anyone else" [2]. Patient-Reported Outcome Measures (PROMs), including surveys, questionnaires and scales have emerged as invaluable instruments for capturing these PROs [3].

These measures are particularly important because patient goals may differ from those of clinicians and researchers [4]. Beyond their role in clinical research, the systematic use of PROMs is also recommended in clinical practice as they allow healthcare providers to gain a more holistic understanding of the patient’s perspective [5] and make more informed clinical decisions, incorporating data directly from the patient to allow personalized treatment options [6, 7].

While we refer to these measures as PROMs, it is essential to distinguish between those patient-related but reported by caregivers or someone who observes the patient in daily life, known as Observer-Reported Outcome Measures (ObsROMs), and those reflecting the caregiver’s quality of life and impact, termed Caregiver-Reported Outcome Measures (CROMs). ObsROMs are particularly useful for patients who cannot report for themselves, like infants or individuals who are cognitively impaired [8].

The importance of integrating these outcome measures (PROMs, CROMs and ObsROMs) becomes even more evident when applied to conditions like Spinal Muscular Atrophy (SMA) and Duchenne Muscular Dystrophy (DMD).

SMA is a debilitating genetic disorder characterized by progressive muscle weakness and atrophy which leads to significant challenges in daily living and quality of life. Similarly, DMD is a genetic disorder characterized by progressive muscle degeneration and weakness due to the absence of dystrophin protein. Understanding the patient’s perspective on these diseases is crucial to fully capture the multifaceted impact of the conditions and their treatments. Traditionally, the clinical evaluation of SMA and DMD has focused primarily on neuromotor symptoms, using standardized clinical scales (Clinician-reported outcomes—ClinROs) to assess motor function. However, current neuromotor scales often fail to address fatiguability and extramotor symptoms such as oropharyngeal dysfunction and gastrointestinal issues, thus missing the full impact of the disease and treatments on patients' lives. To complement clinical evaluation and provide valuable insights into the patients' perspectives, different disease-specific PROMs are recommended to assess how patients feel or function in SMA and DMD [9, 10]. For SMA, critical dimensions to be monitored include fatiguability, swallowing and respiratory function, emotional well-being, social support, pain and discomfort, overall HRQoL and independence [11–13]. In DMD, key HRQoL aspects are motor functionality, psychological well-being, family and social support, independence in daily activities, and pain and fatiguability [14–16].

In the era of disease-modifying therapies, the scientific and clinical communities agree on the necessity of a multidimensional approach to managing these neuromuscular diseases. PROMs can provide a valuable complement to clinical assessments by emphasizing the patient's voice and capturing aspects of health and well-being that clinical tests may overlook [17]. In 2020, eminent authors have gathered to propose a harmonized approach and a roadmap of possible tools to be used in different settings in SMA and DMD [18].

In order to contribute to this field and proceed on the path laid out [17, 18], the objective of this study is to provide an up-to-date overview of the patient-reported or observer-reported instruments developed and/or recommended in clinical research and clinical practice in SMA and DMD. The second part of our study explores the inclusion of PROMs, CROMs, and ObsROMs in clinical research and regulatory processes, focusing on their role as endpoints in study protocols, publications, and approval or reimbursement assessments in Europe and Italy. This analysis examined their application in key therapies for SMA and DMD. The ultimate purpose of this study is to identify gaps, challenges, and opportunities in the integration of these outcome measures into the clinical and regulatory landscapes for SMA and DMD, thereby providing actionable insights to enhance patient-centered approaches in both research and practice.

## Materials and methods

### Repository development

This study is part of the PRO4All project, a multi-stakeholder initiative intended to generate awareness and knowledge around the potential and use of PROs in clinical and regulatory decisions. We aimed to retrieve PROMs, ObsROMs and CROMs in SMA and DMD by searching various sources, starting from the ePROVIDE database (https://eprovide.mapi-trust.org/) using as keywords 'spinal muscular atrophy' and 'duchenne muscular dystrophy'. For each measure identified in ePROVIDE, we analyzed the ‘Basic Description’ scheme and selected those reporting SMA and DMD among the 'Population of development/Disease(s)’ section and 'PRO' or 'Composite COA’ including PRO in the 'Type of Clinical Outcome Assessment (COA)' section.

Additionally, we searched PubMed by using as keywords “patient-reported outcome”, “patient-reported outcome measures”, “caregiver-reported outcome measures”, “spinal muscular atrophy”, “duchenne muscular dystrophy”, “review” to retrieve any undetected instruments in the published literature (last extraction: June 2024).

The instruments identified were categorized as PROMs when used (or recommended) for SMA and DMD patients in either research or clinical practice, as ObsROMs when intended to assess the patient’s health status but reported by someone other than the patient (e.g., a parent) and as CROMs when assessing self-reported caregiver’s health status or caregiving situation. The questionnaire selection and classification were done by one reviewer (FM) and double-checked by another one (OC), with any disagreement solved by consensus.

To perform a content analysis [19, 20], we extracted individual items from either the full-text questionnaire (when freely available) or related publications or websites.

We developed a data extraction form to systematically collect information on each instrument. Using Google Scholar, PubMed, and Scopus, we identified published articles reporting the validation process, psychometric evaluation or Minimal Clinically Important Difference (MCID), type of scoring, and Recall Period determination for individual instruments in SMA and DMD populations. Finally, questionnaire items were extracted and assigned with a specific domain according to a predefined 38-item taxonomy [21] developed for the classification of outcomes included in clinical trials, Core Outcome Sets (COS), systematic reviews, and trial registries to identify the most targeted dimensions by specific SMA and DMD indications. The assignment of domains was performed by one reviewer (FM) and double-checked by the other one (OC), according to a process previously reported [20, 22]. These elements were collected within a repository with the purpose of facilitating the comparison of patient measures and the interpretation of findings for informed decision-making.

### Inclusion of instruments in clinical trials, regulatory documents and innovativeness forms

This comprehensive analysis encompassed:

- Clinical Study Protocols and Publications: We examined pivotal and supportive clinical studies, accessing their protocols via ClinicalTrials.gov (where publicly available) and identifying corresponding primary publications through PubMed searches using trial NCT codes.
- European Regulatory Approval Reports: We investigated European Public Assessment Reports (EPARs) published by the European Medicines Agency (EMA), searching for relevant medicines for human use on their official website (https://www.ema.europa.eu/en/medicines).
- Italian Innovative Reports: For drugs with an innovative designation in Italy, we consulted the public innovative evaluation reports available on the Italian Medicines Agency (AIFA) website.

Our analysis specifically focused on four disease-modifying therapies: nusinersen (Spinraza), onasemnogene abeparvovec (Zolgensma), and risdiplam (Evrysdi) for Spinal Muscular Atrophy (SMA), and ataluren (Translarna) for Duchenne Muscular Dystrophy (DMD) (Fig. 2). Although ataluren was later rejected and is currently not approved by EMA, at the time of our analysis it was still under conditional approval. We chose to include it as a concrete example of how PROMs were utilized in the clinical and regulatory development process. For nusinersen and onasemnogene abeparvovec, which received innovative designation in Italy, we specifically referenced the AIFA innovative evaluation reports.

For all the reviewed documents, we searched for PROs and PROMs using a list of keywords, including: “PRO”, "patient-reported", "patient reported", “quality of life", "QoL", "caregiver", "item”, "domain", "questionnaire", "scale", and "symptom". Additionally, we specifically examined the endpoint paragraphs, main text, and supplemental materials.

We recorded the presence of any PRO/PROM and their relative features: type of clinical endpoint (classification of the PRO/PROM as a primary, secondary, tertiary, exploratory, or other endpoint); type of Clinical Outcome Assessment (specifying whether it was a patient-reported outcome—PRO, caregiver-reported outcome—CRO, or observer-reported outcome—ObsRO); detail description of the specific outcome measured by the PRO/PROM.

## Results

**Fig. 1:**
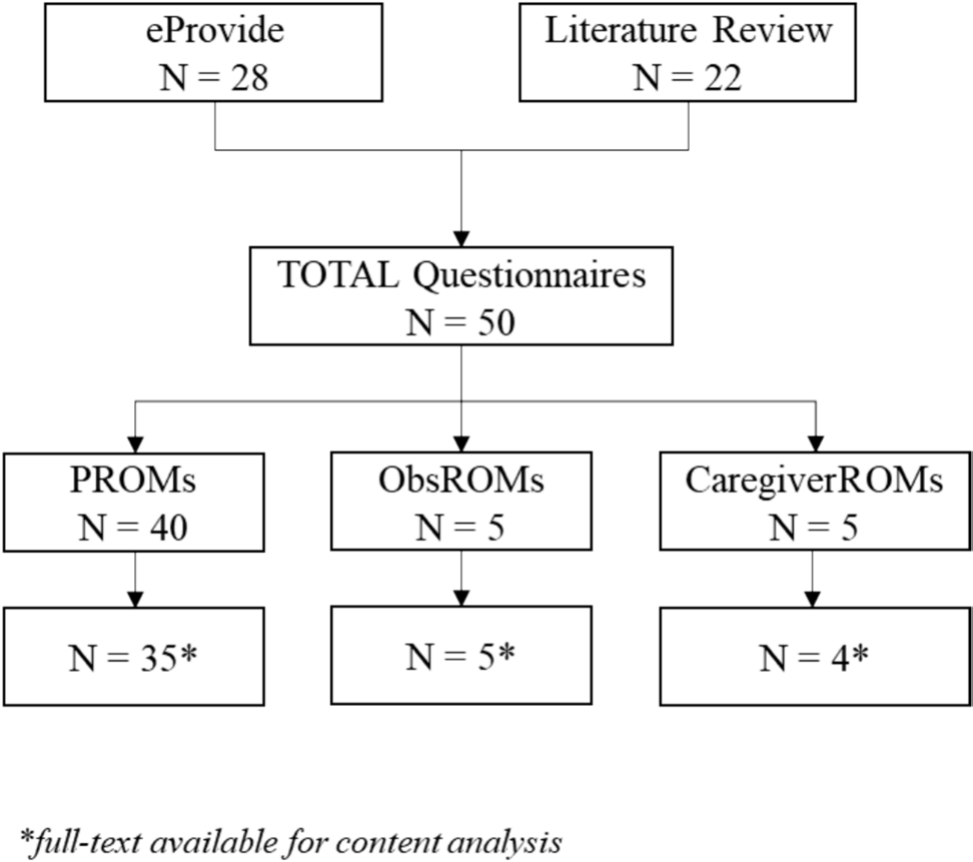
Flowchart of the selection process for SMA and DMD Questionnaires

The initial search identified a total of 28 questionnaires from the ePROVIDE database and 22 from the published literature (Appendix A). Among these, 40 instruments were classified as PROMs, 5 as ObsROMs, and 5 as CROMs (Fig. 1).

### Instrument description

Of the 50 questionnaires identified, 44 (88.0%) were available in full text and included in the analysis, comprising 35 PROMs, 5 ObsROMs, and 4 CROMs. As shown in Table 1, the 35 PROMs are divided into:

- 15 (42.9%) were specifically developed for neuromuscular diseases;
- 4 (11.4%) were specific to DMD:
- the *Pediatric Quality of Life Inventory™ Duchenne Muscular Dystrophy Module* (PedsQL™ DMD Module),
- the *Duchenne Muscular Dystrophy Quality of Life Measure* (DMD-QoL),
- the *Duchenne Muscular Dystrophy Upper Limb Patient-Reported Outcome Measure* (DMD Upper Limb PROM),
- and the *Life Satisfaction Index for Adolescents* (LSI-A);
- 2 (5.7%) were specific to SMA:
- the *Spinal Muscular Atrophy Independence Scale* (SMAIS-ULM, self- and caregiver-reported versions),
- and the *Spinal Muscular Atrophy Functional Rating Scale* (SMAFRS).
- 14 PROMs (40.0%) were designed for the general population but have been recommended or used in studies involving DMD and SMA patients, based on literature or expert consensus.

**Table 1:** Total Questionnaires (N = 44) by therapeutic area

Therapeutic area	PROM (%)	ObsROM (%)	CROM (%)	TOTAL (%)
SMA	2 (5.7)	0 (0.0)	1 (25.0)	3 (6.8)
DMD	4 (11.4)	2 (40.0)	1 (25.0)	7 (15.9)
Neuromuscolar disease	15 (42.9)	2 (40.0)	0 (0.0)	17 (38.6)
General population	14 (40.0)	1 (20.0)	2 (50.0)	17 (38.6)
Total	35 (100.0)	5 (100.0)	4 (100.0)	44 (100.0)

SMA: Spinal Muscular Atrophy, DMD: Duchenne Muscular Dystrophy

Out of the total 44 questionnaires, evidence on psychometrics and/or validation was found for 43 (97.7%), and on MCID determination for 6 (13.6%) only. The recall period was ‘last week’ in about half of the cases (52.3%), and ‘today’ in 6.8%. As for the scoring system, 29 (65.9%) questionnaires had only the total score, while 13 (29.5%) had both total and sub scores. In addition, 5 macro-families of questionnaires were identified: NeuroQol (18.2%), Pediatric Quality of Life Inventory—PedsQoL (8.6%), Kidscreen (8.6%), Patient-Reported Outcomes Measurement Information System -PROMIS (4.5%) and Work Productivity and Activity Impairment Questionnaire—WPAI (4.5%), as shown in Table 2.

**Table 2:** Questionnaires (N = 44) characteristics by type

Validation	PROM (%)	ObsROM (%)	CROM (%)	TOTAL (%)
Yes	35 (100.0)	5 (100.0)	3 (75.0)	43 (97.7)
No	0 (0.0)	0 (0.0)	1 (25.0)	1 (2.3)
**Scoring**				
Only total score	22 (62.9)	3 (60.0)	4 (100.0)	29 (65.9)
Total and sub score	11 (31.4)	2 (40.0)	0 (0.0)	13 (29.5)
Only sub score	2 (5.7)	0 (0.0)	0 (0.0)	2 (4.5)
**Recall period**				
Last week	21 (60.0)	0 (0.0)	2 (50.0)	23 (52.3)
Not specified	5 (14.3)	1 (20.0)	2 (50.0)	8 (18.2)
Not found	3 (8.6)	2 (40.0)	0 (0.0)	5 (11.4)
Today	2 (5.7)	1 (20.0)	0 (0.0)	3 (6.8)
Other	2 (5.7)	1 (20.0)	0 (0.0)	3 (6.8)
Last 4 week	2 (5.7)	0 (0.0)	0 (0.0)	2 (4.5)
**MCID**				
Yes	6 (17.1)	0 (0.0)	0 (0.0)	6 (13.6)
No	29 (82.9)	5 (100.0)	4 (100.0)	38 (86.4)
**Family**				
NeuroQol	8 (22.9)	0 (0.0)	0 (0.0)	8 (18.2)
PedsQL	3 (8.6)	0 (0.0)	0 (0.0)	3 (6.8)
KIDSCREEN	3 (8.6)	0 (0.0)	0 (0.0)	3 (6.8)
PROMIS	2 (5.7)	0 (0.0)	0 (0.0)	2 (4.5)
WPAI	0 (0.0)	0 (0.0)	2 (50.0)	2 (4.5)
No family	19 (54.3)	5 (100.0)	2 (50.0)	26 (59.1)

MCID: Minimal Clinically Important Difference; PedsQL: Pediatric Quality of Life Inventory, PROMIS: Patient-Reported Outcomes Measurement Information System; WPAI: Work Productivity and Activity Impairment questionnaire

## Content analysis

The content analysis was conducted on 955 items extracted from 44 instruments out of the total 50 included (88.0%). The most frequent outcome domain assigned to individual items was ‘physical functioning’ (29.4%), followed by ‘emotional functioning/wellbeing’ (23.3%), social functioning (17.3%), general outcomes’ (e.g., fatigue, malaise, anorexia, pain) (6.1%), and cognitive functioning (3.8%), as shown in Table 3. The mean number of items per questionnaire was 22.6 ± 17.1 (range: 5–86).

**Table 3:** Content analysis: items (N = 955) categorization based on the outcome taxonomy [21] by questionnaire type

Domain	PROM (%)	ObsROM (%)	CROM (%)	Total (%)
Physical functioning	244 (31.8)	49 (30.1)	0 (0.0)	293 (29.4)
Emotional functioning/wellbeing	183 (23.8)	30 (18.4)	19 (29.7)	232 (23.3)
Social functioning	133 (17.3)	22 (13.5)	17 (26.6)	172 (17.3)
General outcomes	57 (7.4)	3 (1.8)	1 (1.6)	61 (6.1)
Cognitive functioning	20 (2.6)	12 (7.4)	6 (9.4)	38 (3.8)
Role functioning	23 (3.0)	4 (2.5)	10 (15.6)	37 (3.7)
Delivery of care	37 (4.8)	0 (0.0)	0 (0.0)	37 (3.7)
Personal circumstances	28 (3.6)	0 (0.0)	2 (3.1)	30 (3.0)
Metabolism and nutrition outcomes	4 (0.5)	17 (10.4)	0 (0.0)	21 (2.1)
Perceived health status	5 (0.7)	8 (4.9)	1 (1.6)	14 (1.4)
Societal/carer burden	6 (0.8)	4 (2.5)	4 (6.3)	14 (1.4)
Musculoskeletal and connective tissue outcomes	9 (1.2)	0 (0.0)	0 (0.0)	9 (0.9)
Respiratory, thoracic and mediastinal outcomes	3 (0.4)	6 (3.7)	0 (0.0)	9 (0.9)
Need for intervention	5 (0.7)	0 (0.0)	4 (6.3)	9 (0.9)
Other	11 (1.4)	8 (4.9)	0 (0.0)	19 (1.9)
Total	768 (100.0)	163 (100.0)	64 (100.0)	995 (100.0)

## Use of patient-, observer-, and caregiver-reported outcome measures in pivotal clinical study protocols, published trial results and regulatory reports in SMA and DMD

### Pivotal clinical study protocols

**Fig. 2:**
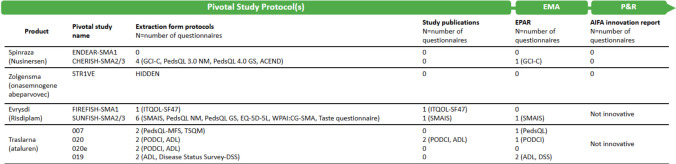
Flowchart of PROM, ObsROM, and CROM Inclusion in Drug Development and Regulatory Assessment for SMA and DMD (n = number of questionnaires). *ACEND: Assessment of Caregiver Experience with Neuromuscular Disease; ADL: Activities of Daily Living; DSS; Disease Status Survey; EQ-5D-5L: EuroQoL-5Dimension-5Livel; GCI-C: Clinical Global Impression – Change; ITQOL-SF47: Infant and Toddler Quality of Life Questionnaire—Short Form; PedsQL- NM|GS|MFS: Pediatric Quality of Life Inventory – Neuromuscular Module|Generic Score|Multidimentional Fatigue Score; PODCI: Pediatric Outcomes Data Collection Instrument; SMAIS: Spinal Muscular Atrophy Independence Scale; TSMQ: Treatment Satisfaction Questionnaire for Medication; WPAI:CG-SM: Work Productivity and Activity Impairment: Caregiver for SMA. EMA: European Medicine Agency; EPAR European Public Assessment Report; AIFA: Italian Medicines Agency, P&R: Price and Reimbursement*

In the pivotal clinical study protocols**,** for nusinersen, onasemnogene abeparvovec, risdiplam, and ataluren, we identified 14 unique instruments, including PROMs, ObsROMs, and CROMs (Fig. 2 and Appendix B). Of the 14 instruments identified, five were specifically designed for SMA or neuromuscular disorders (NMD). These include the Spinal Muscular Atrophy Independence Scale (SMAIS), used in the SUNFISH trial to assess assistance needs in patients with SMA types 2 and 3; the Work Productivity and Activity Impairment: Caregiver for SMA (WPAI:CG-SMA); the Pediatric Quality of Life Inventory-Neuromuscular Module (PedsQL-NM), developed to assess health-related quality of life in NMDs; and the Assessment of Caregiver Experience with Neuromuscular Disease (ACEND), used in the CHERISH study to evaluate caregiver experience. On average, clinical protocols included three instruments per study (range: 1 to 6). Notably, none of these measures were employed as primary endpoints, but were instead classified as secondary, tertiary, or exploratory.

These were largely documented in “the instrument description” section of protocols, except for the Taste Questionnaire, a study-specific tool. Among these, the PedsQL Generic Score was the most frequently included (N = 4), appearing in protocols for both SMA and DMD trials for nusinersen, risdiplam, and ataluren. The PedsQL-NM 3.0 was common (N = 2) to nusinersen and risdiplam trials, serving as generic instruments for pediatric populations. CROMs like the ACEND, WPAI:CG-SMA, and Infant and Toddler Quality of Life Questionnaire—Short Form (ITQOL-SF47) assessed the impact of caregiving on caregiver health and life, while ObsROMs allowed caregivers to report outcomes for patients unable to self-report. Notably, the STR1VE study lacked the outcome measures considered in this study.

#### Study publications

Of the 14 identified measures, only four (28.6%) – ITQOL-SF47, SMAIS, Pediatric Outcomes Data Collection Instrument (PODCI), Activities of Daily Living (ADL) & Disease Status Survey (DSS) – were mentioned in published trial results (Fig. 2 and Appendix B). PROMs, ObsROMs and CROMs were often included as secondary or exploratory endpoints. For risdiplam, the ITQOL-SF47 (in FIREFISH study) assessed quality of life and parental impact, showing changes in median scores without statistical analysis. The SMAIS (in SUNFISH study) demonstrated numerical improvements in caregiver- and patient-reported scores, highlighting reduced caregiver burden and improved patient independence. A follow-up publication confirmed statistically significant improvements in SMAIS scores, reinforcing risdiplam’s effect on upper limb function. For ataluren, the PODCI and ADL survey (in Study 020) showed no significant differences in primary analysis but identified clinically meaningful improvements in specific subgroups for physical functioning domains.

#### EPAR and AIFA reports

Six measures, including CGI-C, SMAIS, PedsQL-GS, PODCI, ADL and DSS were referenced in European Public Assessment Reports (EPARs) for Spinraza, Evrysdi, and ataluren, highlighting partial inclusion in regulatory documentation. The Spinraza EPAR briefly noted Clinical Global Impression – Change (CGI-C) improvements reported by caregivers and investigators, absent from published trial results. In the Evrysdi EPAR, SMAIS data were included with numerical improvements favoring risdiplam, though without statistical significance. Data from the ITQOL-SF47 in FIREFISH were submitted to EMA but excluded from the EPAR due to its non-preference-based nature, limiting its relevance for utility estimation. For ataluren, the Translarna EPARs over a decade of conditional approvals included positive PedsQL Generic Score results (Study 007), PODCI improvements in physical functioning (2016 EPAR), and retrospective survey data showing improvements in 34% of patients (2019 EPAR). However, planned analyses for some measures were not conducted due to insufficient baseline data.

None of these PROMs were cited in AIFA’s innovation designation documents for Spinraza and Zolgensma (Fig. 2).

## Discussion

This study provides an overview of the use of PROMs, ObsROMs, and CROMs in clinical and regulatory contexts for SMA and DMD by analyzing the content and characteristics of existing tools, which often focus on well-established constructs (e.g., physical functioning) but still present significant gaps and limitations.

Specifically, the analysis of the repository showed that while there is a wide array of PROMs available, many are not adequately tailored to neuromuscular disorders and only 6 (13,6%) are disease-specific. Generic tools, such as the widely used PedsQL, are criticized for their lack of specificity and limited applicability to pediatric neuromuscular populations [22]. Furthermore, challenges related to recall period selection and the definition of MCID represent two distinct yet critical barriers to the interpretability of PROMs. The former compromises the reliability of patient-reported data, while the latter undermines the identification of clinically meaningful change.These limitation emphasizes the necessity for either the rigorous development of new instruments or the adaptation and validation of existing ones. Current initiatives, such as those by Mercuri et al. [18] and the SMA-TOOL study [23], aim to address gaps in assessing fatigue, swallowing, and other critical dimensions.

Moving from instrument availability to their practical application, the analysis of the clinical trial protocols demonstrated that the outcome measures considered in this study were widely included as exploratory or secondary endpoints. However their representation in regulatory reports and publications was sparse: only six of the 14 (42.4%) instruments identified were mentioned in regulatory documents like EMA’s EPARs, and 4 (28,6%) were discussed in trial publications. For example, while SMAIS was consistently included across risdiplam evaluations, other PROMs were excluded in reports, signaling a lack of standardization and prioritization in their use [18]. Regulatory agencies often appear to sideline PROMs in decision-making processes, despite their potential to capture patient-centered outcomes. However, at European level the recent reflection paper on patient experience data produced by EMA should be welcomed as a positive step in the right direction [24]. This study demonstrates that while the inclusion of PROMs in clinical research and regulatory contexts is gaining traction, their implementation remains suboptimal. To address these operational and methodological challenges,electronic PROMs (ePROMs) systems could represent a promising avenue for improving data collection and validity, especially for caregiver-reported outcomes in pediatric settings, although barriers such as costs and technological accessibility must be overcome.This underscores the need for reforms, such as those proposed by the PRO4ALL initiative, which advocates for integrating PROMs systematically across clinical and regulatory workflows [25]. By aligning with ongoing updates in AIFA’s organizational structure and regulatory strategy, the initiative aims to bridge the gap between patient involvement and standardized data, ensuring that patient perspectives inform decision-making and improve healthcare outcomes.Finally, the findings call for a collaborative effort among stakeholders to ensure that PROMs fully contribute to the assessment of therapeutic outcomes, particularly in rare diseases such as SMA and DMD [26, 27].

## Supplementary Information

Below is the link to the electronic supplementary material.

Supplementary file1 (XLSX 23 KB)

Supplementary file2 (XLSX 69 KB)
